# Prevalence of Neuroendocrine Tumours (NET) in Patients Undergoing Appendicectomy for Acute Appendicitis: A Tertiary Care Study

**DOI:** 10.7759/cureus.50783

**Published:** 2023-12-19

**Authors:** Sreekanthan Gobishangar, Sivakumaran Gobinath, Christine Thevamirtha, Senathiraja Sarmila, Sittampalam Kasthuri, Shathana Paramanathan

**Affiliations:** 1 Surgery, University of Jaffna, Jafffna, LKA; 2 Surgery, Jaffna Teaching Hospital, Jaffna, LKA; 3 Surgery, University of Jaffna, Jaffna, LKA; 4 Health Sciences, Management and Science University, Kula Lampur, MYS

**Keywords:** hemicolectomy, appendiceal neoplasms, appendicectomy, appendicitis, neuroendocrine tumour

## Abstract

Introduction

Acute appendicitis is one of the common causes of acute abdomen in adults, which is managed widely with appendicectomy. Neuroendocrine tumours are the most common appendiceal tumours diagnosed incidentally on appendicectomy specimens.

Methods

Demographic data, presenting complaints, indications for appendicectomy, and the histology findings based on histopathological reports of the patients who have undergone appendicectomy for appendicitis at surgical units of Teaching Hospital, Jaffna, from 1st of January 2019 to 31st of December 2022 were retrospectively analyzed.

Results

Of the 1341 histopathology reports, 0.2% (n=3) were neuroendocrine tumours (NET). The mean age of the patients with NET was 48.6, and 66.6% of them were females. All three NETs identified in appendicectomy specimens were well differentiated and smaller than 2 cm. All three had negative resection margins and were managed only with appendicectomy.

Conclusion

NETs of the appendix are the commonest appendiceal neoplasms. The majority of them are diagnosed incidentally in appendicectomy specimens. Surgical management of the tumours is either by appendicectomy or hemicolectomy, which depends mainly on tumor size. Surgical decisions should be tailor-made to the patients based on multi-disciplinary team decisions.

## Introduction

Acute appendicitis is a common cause of acute abdomen in adults with an average lifetime risk of 7-8% [1]. The incidence of appendicitis in the USA has been reported between 82 and 111 per 100,000 population per year, with a lifetime risk of 6.7% [2]. It is commonly treated with appendicectomy [3]. The highest incidence of appendicitis occurs in the second and third decades of life [4]. The pathophysiology of acute appendicitis is due to obstruction of the lumen by faecoliths, lymphoid hyperplasia, impacted stool, parasitic infection, and rarely by an appendiceal or caecal malignancy. The lumen blockage leads to increased intramural pressure, affecting venous and lymphatic outflow and impaired blood flow and ischemia. The inflammatory process can result in perforation, abscess formation, and generalized peritonitis [5].

Neuroendocrine tumours (NET) are slow-growing tumours arising from enterochromaffin cells of the gastrointestinal tract and the bronchopulmonary system. The appendix is a common site for gastrointestinal carcinoid tumours [6]. Appendicular neuroendocrine neoplasms are sporadic, with a reported annual incidence of 0.4 to 0.6/100,000 in the United Kingdom [7]. NET represents 60% of all appendicular tumours, making it the most common tumor type affecting the appendix [8, 9].

Neuroendocrine tumours of the appendix are usually incidental histological findings in appendicectomy specimens. The reported incidence of neuroendocrine tumours of the appendix in the histology of appendicectomy specimens ranges from 3 to 5 per 1000 specimens [10]. Preoperative clinical diagnosis is difficult unless the patient presents with symptoms and signs of carcinoid syndrome [11]. There is no reported incidence of incidental neuroendocrine tumours of the appendix diagnosed in appendicectomy specimens in Sri Lanka.

This study aims to determine the prevalence of neuroendocrine tumors (NET) in patients undergoing appendicectomy for acute appendicitis in Teaching Hospital Jaffna and retrospectively analyses the prevalence, mode of treatment, and outcome of neuroendocrine tumours of the appendix incidentally diagnosed from histology of appendix specimens following appendicectomy for appendicitis. 

## Materials and methods

This retrospective descriptive study was conducted at Teaching Hospital, Jaffna, Sri Lanka. It analyzed data of patients who had undergone appendicectomy for appendicitis at surgical units of Teaching Hospital, Jaffna, from the 1st of January 2019 to the 31st of December 2022. Consecutive sampling techniques were used. After seeking ethical approval from the Ethical Review Committee, Teaching Hospital, Jaffna (Reference No: SO4/01/2023), the data were collected retrospectively from the histopathology reports at the Department of Histopathology. The additional data regarding the patient details were collected from electronic health records as well as bed head tickets. Data were collected using a data entry sheet, which includes demographic data, presenting complaints, indication for appendicectomy, and histology findings. All the patients who underwent appendicectomy following a clinical diagnosis of appendicitis were included in the study. The incidence of neuroendocrine tumours of the appendix diagnosed incidentally from histology specimens was calculated as a percentage. The follow-up management and the outcome of those patients were analyzed.

The data collected was analyzed using SPSS version 26 (IBM Inc., Armonk, New York). The demography of the participants was presented in graphs, tables, percentages, mean and standard deviation, and frequencies. A p-value of <0.05 was considered statistically significant.

## Results

**Figure 1:**
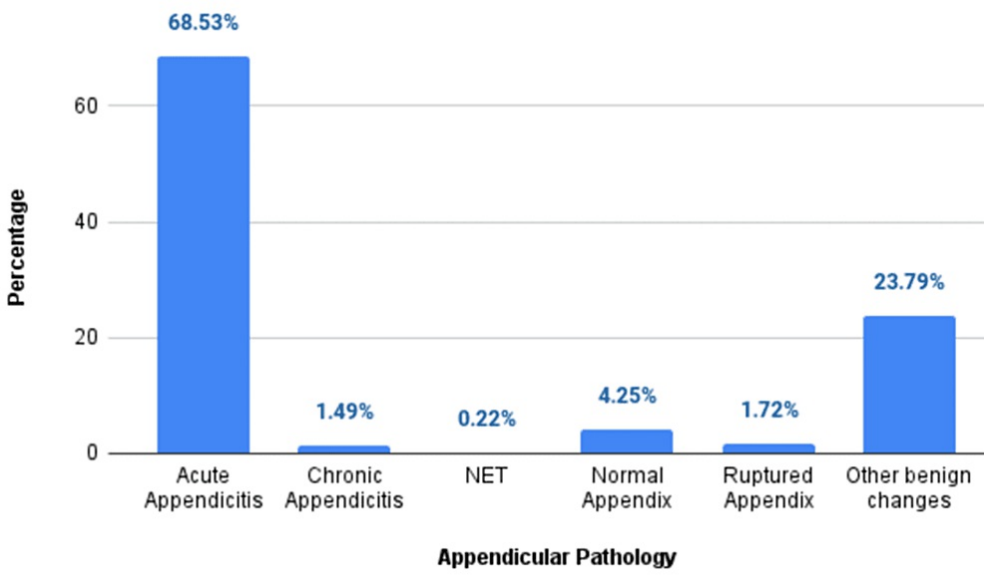
Histopathological findings of the appendicectomy specimens

A total of 1341 appendicectomy specimens of patients with acute appendicitis were analyzed. Out of that, 68.53% (n=919) were histologically acute appendicitis, 23.79% (n=319) had other benign changes (including mucosal lymphoid hyperplasia, fibrous obliteration of the appendix, submucosal lipomatosis, and appendix with a luminal fecalith), 4.25% (n=57) were normal appendix, 1.72% (n=23) were ruptured appendix, 1.49% (n=20) were chronic appendicitis, and 0.22% (n=3) were neuroendocrine tumours (NET) (Figure 1).

The mean age of patients with NET was 48.6 years, and 66.6% of them were females. All three NETs identified in appendicectomy specimens were well differentiated. Two were less than 1 cm in size (4 and 7 mm), and one was 1.5 cm with a mean length of 8.3 mm. Two tumours were confined to the tip of the appendix, and the largest was limited to the wall. One had invaded muscularis propria and two into the subserosal layer with mesoappendicular invasion. One of them had a lymphovascular invasion. 

**Figure 2:**
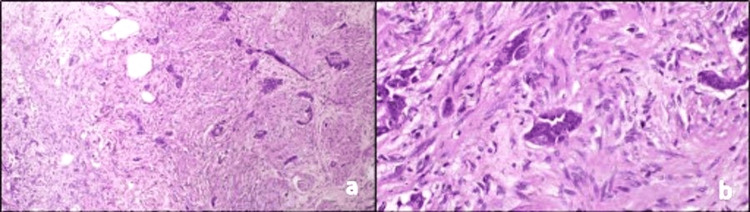
Histopathology images of case one under magnification a) x100 and b) x400 (synaptophysin and chromogranin A stained)

**Figure 3:**
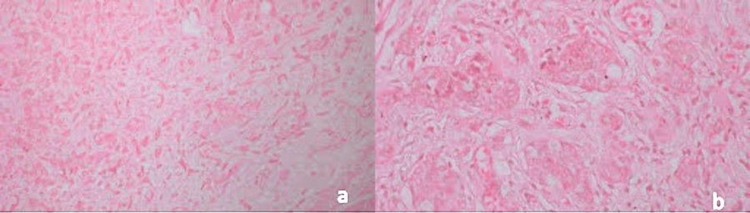
Histopathology images of case three under magnification a) x100 and b) x400 (synaptophysin and chromogranin A stained)

The Ki-67 index of all three tumours was 1%. Synaptophysin and chromogranin A immunomarkers were strongly positive in all the tumours (Figures 2, 3). All tumours were grade 1. All three had negative resection margins, and no further surgical intervention was warranted (Table 1). For the first year, follow-up assessments were conducted every three months. Throughout this period, CT scans were performed for all patients, and after one year of follow-up, no second malignancies or other complications were identified. In the second year, follow-up will be conducted every six months. The follow-up will persist for a duration of ten years, with annual CT scans planned. Any complications that may arise will be recorded. 

**Table 1:** Histopathological findings of the well-differentiated NET of the appendix NET - neuroendocrine tumor

Case	Operative findings	Tumor type	Tumor size	Tumor grade	Ki-67 index	Tumor stage	Tumor site	Lymphovascular invasion	Resection marginal status
1	Inflamed appendix	Well-differentiated NET	4 mm	G1	1%	pT1NxMx	Tip of the appendix, Infiltrates muscularis propria	Not seen	Not involved by the tumor
2	Inflamed appendix	Well-differentiated NET	15 mm along the long axis	G1	1%	pT3NxMx	Confined to the appendiceal wall, infiltrates subserosal layer.	Not seen	<1 mm clearance is present
3	Inflamed appendix	Well-differentiated NET	7 mm	G1	1%	pT3NxMx	Tip of the appendix, infiltrates the subserosal layer.	Present	Base free

## Discussion

Appendiceal neoplasms are broadly divided into epithelial and nonepithelial lesions. Epithelial lesions include mucinous neoplasms and adenocarcinomas; nonepithelial lesions include neuroendocrine tumours (NET) [12]. More than 80% of appendiceal neuroendocrine tumours (NETs) are diagnosed incidentally in appendectomy specimens [13]. Unlike other neuroendocrine tumours of the gut, appendiceal NETs are diagnosed at fairly younger ages in the third to fourth decades of life, which coincides with the mean age of our study. The low incidence of the appendiceal NETs described in the literature is like our study, which is 0.23% [10, 14, 15].

Most of the appendicular NETs were diagnosed at the tip of the appendix, 66% of the specimens. Similar findings have also been described in the literature [12, 16].

The tumor size can best predict clinical behavior and prognosis. In approximately 95% of cases, the tumor size is less than 2 cm, and these tumours are less likely to metastasize [13, 17, 18]. All the patients in our study had tumor sizes less than 2 cm.

Ki67 index can be used to grade the appendiceal Nets. Tumours with a KI 67 index of less than 2% are considered G1, 3-20% are considered G2, and >20% as G3 [19], [20]. In our study, all the tumours were low-grade (G1).

Surgical management of appendiceal NETs can be a simple appendicectomy or right hemicolectomy with lymph node clearance. There are controversies among the guidelines, and the management should be tailored to the patient [21, 22]. As per the North American Neuroendocrine Tumour Society (NANETS) consensus guidelines, small (≤1 cm) well-differentiated carcinoids confined to the tip of the appendix that is completely excised can be regarded as cured if there is no evidence of lymphovascular invasion or invasion into the mesoappendix. A right hemicolectomy is recommended for patients who have appendiceal carcinoids with evidence of tumor invasion at the base of the appendix, patients with tumor size more than 2 cm, in whom size cannot be determined, those with incompletely resected tumours, those with evidence of lymphovascular invasion, those with the invasion of the mesoappendix, patients with intermediate- to high-grade tumours (G2 or G3) and for those with mixed histology [16, 23, 24].

Per the European Neuroendocrine Tumour Society (ENETS) Consensus Guidelines, a well-differentiated appendiceal NET <2 cm is recommended to be cured by appendicectomy, independent of the tumor's location. A right hemicolectomy is justified in those with tumours measuring 1-2 cm but with positive or unclear margins or with mesoappendiceal invasion, higher proliferation rate and/or vascular invasion. Tumours with a diameter >2 cm should be treated with right hemicolectomy [20, 25].

NCCN recommends a simple appendicectomy for tumours less than 2 cm and a right hemicolectomy for those with tumor size >2 cm, with positive resection margins/nodes and incomplete resection margins [23].

In all three of our patients, appendicectomy was considered adequate as two had less than 1 cm tumor size with low grade and low stage, and the third one had a tumor size of 1.5 cm with negative resection margin without lymphovascular invasion and low grade. According to NANET consensus guidelines, the third patient should have undergone a right hemicolectomy as he had mesoappendicular invasion, but it has not been performed. It is also widely considered that right hemicolectomy will not improve survival benefits much in this tumor, affecting the patient's quality of life [21, 26].

Anatomical imaging staging is performed to detect nodal and distant metastases. According to the most recent guidelines, a workup using a triple-phase, contrast-enhanced computed tomography (CT) scan or magnetic resonance imaging (MRI) is advised for patients with an appendiceal NET greater than 2 cm, incomplete resection and positive nodes or margins [23, 27].

Tumours less than 1 cm in size with no aggressive features, which were treated with appendectomy with no residual disease, do not need active surveillance. Surveillance is also not required for tumours between 1 and 2 cm in size, which have undergone right hemicolectomy with no residual disease. NANET guidelines recommend against surveillance imaging for well-all NETs less than 2 cm in size. However, ENETS recommends follow-up for patients only with tumours measuring 1-2 cm with residual disease, lymph node involvement, lymphovascular invasion, and higher tumor grade [23, 24, 28, 29].

The large number of patients included is the main strength of the study. However, a major limitation of this study is its retrospective design. This can be solved by conducting a prospective study in the near future. Also, the study was conducted in a single tertiary care center. Thus, there is a possibility of unintentional patient selection bias.

## Conclusions

NETs of the appendix are the most common appendiceal neoplasms. The majority of them are incidental diagnoses in appendicectomy specimens. Therefore, it is recommended that a histological assessment of all appendicectomy specimens be done. In this present study, one case exhibited invasion into the muscularis propria, while two cases showed invasion into the subserosal layer with mesoappendicular involvement. Additionally, one of these cases demonstrated lymphovascular invasion. Surgical management of the tumours depends on the size, grade, and stage. It is widely accepted that a simple appendicectomy is adequate for a tumor size less than 2 cm and a right hemicolectomy for a tumor size more than 2 cm. Although various guidelines suggest different indications for hemicolectomy, patient management should be tailored and based on multidisciplinary team decisions. As the study was done retrospectively in a single center, we will conduct a prospective study in a multicenter in the near future.
